# Relation between phalangeal bone mineral density and radiographic knee osteoarthritis: a cross-sectional study

**DOI:** 10.1186/s12891-016-0918-x

**Published:** 2016-02-11

**Authors:** Zhen-han Deng, Chao Zeng, Yu-sheng Li, Tuo Yang, Hui Li, Jie Wei, Guang-hua Lei

**Affiliations:** Department of Orthopaedics, Xiangya Hospital, Central South University, No.87 Xiangya Road, Changsha, Hunan Province 410008 China; Health Management Center, Xiangya Hospital, Central South University, Changsha, Hunan Province 410008 China; Department of Epidemiology and Health Statistics, School of Public Health, Central South University, Changsha, Hunan Province 410008 China

**Keywords:** Osteoarthritis, Knee, Phalangeal, Bone mineral density, Kellgren–Lawrence grade

## Abstract

**Background:**

Major reports have suggested that bone mineral density (BMD) is higher in patients with osteoarthritis (OA), while other studies do not agree. Our aim was to examine the cross-sectional association between phalangeal BMD and radiographic knee OA.

**Methods:**

A total of 2855 participants were included in this study. Radiographic knee OA was defined as Kellgren-Lawrence (K-L) Grade ≥ 2 in at least one leg. BMD scans of the middle phalanges of the second, third and fourth digits of the nondominant hand were performed with a compact radiographic absorptiometry system (Alara MetriScan®). A multivariable logistic analysis model was applied to test the relation between phalangeal BMD with radiographic knee OA, the presence of knee osteophytes (OSTs), and knee joint space narrowing (JSN) after adjusting for a number of potential confounding factors.

**Results:**

The multivariable-adjusted odds ratios with 95 % confidence intervals [ORs (95 % CI)] of radiographic knee OA across phalangeal BMDs were 1.08 (95 % CI 0.89–1.32) and 0.62 (95 % CI 0.45–0.86), respectively. The P for trend was 0.09. For the female population, the multivariable-adjusted ORs (95 % CI) of radiographic knee OA across phalangeal BMD were 1.01 (95 % CI 0.73–1.37) and 0.58 (95 % CI 0.38 − 0.87), respectively. The P for trend was 0.02. This positive finding, however, did not exist in the male subgroup. There was a significantly lower prevalence of OST in the osteoporosis (OP) group than in the normal group (OR = 0.59, 95 % CI 0.40–0.88; P for trend was 0.01). In contrast, the prevalence of JSN was significantly higher in the osteopenia group (OR = 1.22, 95 % CI 1.00–1.48) and the OP group (OR = 1.35, 95 % CI 1.00–1.84) than in the normal group. The P for trend was 0.02.

**Conclusions:**

This study observed lower odds for the presence of radiographic knee OA and OST in OP patients than in normal subjects. The prevalence of JSN was higher in the osteopenia and OP groups than in normal subjects.

**Electronic supplementary material:**

The online version of this article (doi:10.1186/s12891-016-0918-x) contains supplementary material, which is available to authorized users.

## Background

Osteoarthritis (OA), the most prevalent joint disease, is characterized by progressive loss of articular cartilage, which leads to chronic pain and functional restrictions in affected joints. Worldwide estimates are that about 10 % of men and 18 % of women and about 60–65 % of those > 60 years of age have symptomatic OA and 80 % of them have movement limitations [1]. Likewise, the prevalence of .osteoporosis (OP) increases with age. OP is a metabolic bone disease characterized by abnormalities in the amount and microarchitectural arrangement of bone tissue that could lead to enhanced bone fragility and a consequent increase in fracture risk [2]. The lifetime risk of a hip fracture from age 50 years onward has been estimated at 17 % for white women and 6 % for white men in the United States [3]. The association between OA and OP has remained controversial since the release of the first research outcome 40 years ago, which indicated an apparent inverse relation between these two common diseases [4]. To date, no consensus has been reached.

Both OA and OP are multifactorial diseases and are influenced by genetics, external environment, the endocrine system, metabolism, biomechanics and trauma [5–9]. Although OA and OP are generally considered different diseases, they share several of the same risk factors, such as bone, cartilage metabolism, aging, sex, and timing of menopause [8–12]. These two common age-related disorders can cause impairment of activities of daily life and quality of life, leading to increased morbidity and mortality among the elderly.

The advent of precise, accurate measurements of bone mass and density has given researchers tools to examine the relation between OA and OP relative to bone [13]. Bone mass change is reflected in bone mineral density (BMD). According to the World Health Organization (WHO), BMD measurement has been considered the gold standard for diagnosing OP [14]. Patients with OA have been considered to have an elevated BMD [15–20], as it has long been hypothesized that systemic or local bone BMD is involved in the pathogenesis of cartilage degradation [21, 22]. Previous studies have indicated that patients with OA of the knee were more likely to have greater bone mass than normal subjects or those with OP. In fact, their BMD was found to be elevated in the spine [23, 24], femoral neck [22, 25], forearm [26, 27], and knee [28, 29]. Other studies, however, indicated that, despite a higher than average bone mass, women with greater joint OA do not have the reduced risk of fracture that a higher bone mass should confer [30, 31]. Some even found lower BMD at the affected sites [32, 33]. These arguments lead to the assumption that the relation between BMD and OA is complicated and may differ by sites or stages. Based on measurements of phalangeal BMD and radiographic knee OA in a large number of apparently healthy Chinese subjects, this cross-sectional study aimed to examine whether the positive association between phalangeal BMD and radiographic knee OA exists in a Chinese population.

## Methods

### Study population

The Xiangya Hospital Health Management Center Study (XYHMCS) included a cohort consisting mainly of apparently healthy Chinese people from the general public who were undergoing health screening. The study design has been published elsewhere [34]. Data were collected from 2855 participants (1609 men, 1246 women) who voluntarily underwent a routine comprehensive health checkup at the Department of Health Examination Center Xiangya Hospital, Central South University in Changsha, Hunan Province, China from October 2013 to July 2014. Registered nurses interviewed all participants during the examination process using standard questionnaires, aiming to gather information on demographic characteristics and health-related habits. Inclusion criteria were as follows: (1) ≥ aged 40 years; (2) were members of general public; (3) had undergone the weight-bearing bilateral anteroposterior radiography of the knee and non-dominant hand BMD test; (4) had completed the semi-quantitative Food Frequency Questionnaire (FFQ) about the average consumption of foods and drinks over the past year; (5) had all basic characteristics, such as age, sex, body mass index (BMI), and smoking status. Initially, this cross-sectional study included 4622 participants who underwent weight-bearing bilateral anteroposterior radiography of the knee and no-dominant hand BMD test. Then, we excluded all individuals with radiographic evidence of other joint diseases such as osteochondroma or fracture (*n* = 105), those who did not complete the FFQ (*n* = 1528), and those < 40 years (*n* = 134).

The ethics committee of Xiangya Hospital of Central South University approved this study. All participants gave written informed consent at the time of recruitment.

### Radiographic assessment of knee OA

Subjects had weightbearing semi-flexed posteroanterior and lateral view knee radiographs obtained using a standard, validated protocol [35, 36]. Two orthopedists, without knowledge of the participants’ clinical symptoms independently assessed all radiographs using the Kellgren − Lawrence (K − L) radiographic atlas [37]. Disagreements between the two orthopedists were resolved by discussion. If at least one knee joint was graded as K − L ≥ 2, the participant was diagnosed with radiographic knee OA.

Interrater reliability was calculated based on 40 random radiographs assessed by two orthopedists. Intrarater reliability was calculated based on the 40 random radiographs measured by one orthopedist, with each radiograph assessed twice, independently. The reliability of the measurements were examined using the kappa (κ) test. Interrater and intrarater reliability were both satisfied (κ = 0.85 and 0.68, respectively). In addition, joint space narrowing (JSN) and osteophytes (OSTs) were assessed individually based on a scale of 0–3 (0 = normal, 3 = most severe) according to the Osteoarthritis Research Society International atlas [38]

### BMD measurement

BMD measurement is a universal method for arriving at an early diagnosis of OP. In this study, BMD was measured at the middle phalanges of the second to fourth fingers on the no-dominant hand using a compact radiographic absorptiometry system (Alara MetriScan®; Alara Inc., Fremont, CA, USA). Participants were asked to place the non-dominant hand on the molded support plate with all ornaments removed. BMD was recorded in arbitrary units (mineral mass/area) and in grams per square centimeter (g/cm^2^).

our staff only recorded data of people who are osteopenia or OP in order to inform patients their illness. But the data of normal BMD people wasn’t recorded.

T-scores were calculated based on the group ages according to a standard BMD database provided by the manufacturer containing data on healthy controls and ages in years. Specifically, T-scores were derived from the reference database by comparing the measured BMD with the average BMD for healthy subjects of the same sex and age. According to the WHO, BMD levels within 1 standard deviations (SD) of a normal young adult are considered normal. Osteopenia (low bone mass) refers to the condition where the BMD level is 1.0–2.5 SD below that of a normal young adult. OP refers to the condition where the BMD level is ≥ 2.5 SD below normal [39].This peripheral densitometry device has advantages, including low cost, efficiency, portability and low X-ray dose (<0.02 μSV per examination), making it suitable for epidemiological screening. The mean coefficient of variation of this method was 1.7 % [40]. Normal BMD normal is defined as a BMD within 1 SD of the young adult reference mean [14]. In this study, the control group was defined as the reference.

### Assessment of dietary and non-dietary exposures

A semi-quantitative FFQ (SFFQ) especially designed for the population in Hunan province of China was used to evaluate dietary intake. This SFFQ contains 63 food items that are commonly consumed in Hunan province. Participants were required to answer the frequency—never, once per month, two to three times per month, one to three times per week, four to five times per week, once per day, twice per day, or three times or more per day—they consumed each food item and the average amount they consumed for each time (<100 g, 100–200 g, 201–300 g, 301–400 g, 401–500 g, ≥ 500 g) during the past year. To help them make choices more easily and accurately, colored pictures showing food samples with labeled weights were provided. The validity of the SFFQ was tested by comparing it with the 24-h dietary recall method for a similar population. The Chinese Food Composition Table was referenced to calculate the individual composition of macronutrients and micronutrients of the included foods [41]. This SFFQ has been validated and was used in a previously published study [42]. The weight and height of each subject were measured to calculate the BMI. People with BMI ≥ 25 kg/m^2^ were defined as overweight. [43] The smoking status, alcohol drinking status, and use of calcium supplements were established by direct face-to-face questioning.

### Statistical analysis

The continuous data were expressed as means ± SD, and the categorical data were expressed in percentages. The OP status was classified into three levels: normal, osteopenia and OP. Differences in continuous data were evaluated by the one-way classification analysis of variance (normally distributed data) or the Kruskal − Wallis H test (non-normally distributed data). Differences in qualitative data were assessed by the *χ*^2^ test. The relation between OA and BMD was determined by the liner trend *χ*^2^ test, and evaluated using multivariable logistic regression. Associations between each OP status and OA were adjusted for the following variables: sex, age, BMI, smoking status, alcohol drinking status, activity level, mean total energy intake, mean Ca intake, and Ca supplementation. A test for linear trend was conducted by putting the BMD status as ranked data into the Logistic regression model. All statistical analyses were performed in both subgroups stratified by sex.

Associations between phalangeal BMD and JSN, phalangeal BMD and OST, and OP status and OA were also evaluated by conducting a multivariable logistic regression. Data analyses were performed using SPSS 17.0 (SPSS, Chicago, IL, USA). *P <* 0.05 was considered to indicate statistical significance.

## Results

Tables 1 and 2 present the basic characteristics of the study population according to their OP and OA status. The overall prevalence of radiographic knee OA of the subjects in this cross-sectional study (aged ≥40 years) was 29.2 % (30.0 % in men, 28.1 % in women). Significant differences were observed across different BMD statuses for age, sex ratio, BMI, smoking status, alcohol drinking status, mean total energy intake, mean Ca intake, and use of Ca supplements.

**Table 1:** Characteristics among 2855 participants according to status of OP

Characteristics	Status of OP			P
	Normal	Osteopenia	OP	
N	1803	786	266	-
OA (%)	26.8	34.2	30.1	0.01
Age (years)	50.6 ± 6.3	54.4 ± 7.4	59.5 ± 6.8	<0.001
Female (%)	36.5	45.5	86.5	<0.001
BMI (kg/m^2^)	24.7 ± 3.4	24.3 ± 3.1	23.6 ± 3.1	<0.001
Overweight (%)	44.0	38.3	30.5	<0.001
Smoking (%)	28.1	25.2	7.9	<0.001
Alcohol drinking (%)	42.1	32.3	16.2	<0.001
Mean total energy intake (kcal/d)	1664.1 ± 774.2	1600.7 ± 732.4	1408.4 ± 546.6	<0.001
Mean Ca intake (kcal/d)	507.9 ± 346.0	492.6 ± 308.4	431.8 ± 253.8	<0.001
Ca supplementation (%)	24.3	28.4	47.0	<0.001

*N* number, *OA* osteoarthritis, *OP* osteoporosis, *BMI* body mass index, *Ca* calcium

**Table 2:** Characteristics among 2855 participants according to status of OA

Characteristics	Status of OA		P
	Normal	OA	
N	2022	833	-
OP (%)	9.2	9.6	0.74
Age (years)	51.4 ± 6.7	55.1 ± 7.8	<0.001
Female (%)	44.3	42.0	0.26
BMI (kg/m^2^)	24.5 ± 3.2	24.5 ± 3.2	0.87
Overweight (%)	40.8	42.0	0.55
Smoking (%)	25.6	24.8	0.67
Alcohol drinking (%)	37.4	36.0	0.49
Mean total energy intake (kcal/d)	1628.1 ± 725.2	1610.1 ± 800.3	0.64
Mean Ca intake (kcal/d)	498.9 ± 342.2	490.9 ± 294.5	0.82
Ca supplementation (%)	26.3	30.6	0.02

*N* number, *OA* osteoarthritis, *OP* osteoporosis, *BMI* body mass index, *Ca* calcium

Significant differences were observed between different OA status levels for age and Ca supplements use. Univariate analysis indicated that there was a significant association between phalangeal BMD and OA (p = 0.01). The multivariable model was adjusted for sex, age, BMI, smoking status, alcohol drinking status, activity level, mean total energy intake, mean Ca intake, and Ca supplementation. The multivariable-adjusted odds ratios with 95 % confidence interval [OR (95 % CI)] of radiographic knee OA across phalangeal BMD were 1.08 (95 % CI 0.89–1.32) and 0.62 (95 % CI 0.45–0.86), respectively. The P for trend was 0.09 (Table 3). For the female subgroup, the multivariable–adjusted ORs (95 % CI) of radiographic knee OA across phalangeal BMD were 1.01 (95 % CI 0.73–1.37) and 0.58 (95 % CI 0.38–0.87), respectively (Table 3). The P for trend was 0.02. This positive finding, however, did not exist in the male subgroup. Furthermore, for the BMI < 25 kg/m^2^ subgroup, the multivariable-adjusted ORs (95 % CI) of radiographic knee OA across phalangeal BMD were 0.97 (95 % CI 0.76–1.25) and 0.56 (95 % CI 0.37–0.84), respectively (Table 3). The P for trend was 0.03. This positive finding, however, did not exist in the BMI ≥ 25 kg/m^2^ subgroup.

**Table 3:** Multivariable-adjusted relations of phalangeal BMD and radiographic knee OA (*n* = 2855)

	Status of OP			
	Normal	Osteopenia	OP	*P* for trend
Participants (n)	1803	786	266	-
Knee OA (n)	484	269	80	-
Multivariable-adjusted OR	1.00 (Reference)	1.08(0.89–1.32)	0.62(0.45–0.86)	0.09
Male subgroup				
Multivariable-adjusted OR	1.00 (Reference)	1.14(0.89–1.46)	0.74(0.34–1.59)	0.67
Female subgroup				
Multivariable-adjusted OR	1.00 (Reference)	1.01(0.73–1.37)	0.58(0.38–0.87)	0.02
BMI < 25 kg/m^2^ subgroup				
Multivariable-adjusted model	1.00 (Reference)	0.97(0.76–1.25)	0.56(0.37–0.84)	0.03
BMI ≥ 25 kg/m^2^ subgroup				
Multivariable-adjusted OR	1.00 (Reference)	1.26(0.93–1.70)	0.78(0.45–1.34)	0.84

*OA* osteoarthritis, *OP* osteoporosis, *BMI* body mass index

*The multivariable-adjusted model was adjusted for sex, age, BMI, smoking status, alcohol drinking status, activity level, mean total energy intake, mean Ca intake, as well as Ca supplementation. The prevalence of osteopenia and OP in overweight (BMI ≥ 25 kg/m2) population was 25.6 % and 6.9 %, respectively

Multivariable-adjusted relations of phalangeal BMD with JSN and OST are show in Table 4. There was a significantly lower prevalence of OSTs in the OP group than in the no-OP group (OR = 0.59, 95%CI 0.40–0.88; P for trend was 0.01). In contrast, the prevalence of JSN was significantly higher in the osteopenia and OP groups than in the no-OP group (osteopenia: OR = 1.22, 95 % CI 1.00–1.48; OP: OR = 1.35, 95 % CI 1.00–1.84). The P for trend was 0.02.

**Table 4:** Multivariable-adjusted relations of phalangeal BMD with JSN and OST (*n* = 2855)

	Status of OP			
	Normal	Osteopenia	OP	*P* for trend
Participants (n)	1803	786	266	-
OST (n)	195	122	60	-
Multivariable-adjusted OR	1.00 (Reference)	0.85(0.65, 1.12)	0.59(0.40, 0.88)	0.01
JSN (n)	444	245	102	-
Multivariable-adjusted OR	1.00 (Reference)	1.22 (1.00, 1.48)	1.35 (1.00, 1.84)	0.02

*OP* osteoporosis, *OST* Osteophyte, *JSN* joint space narrowing

*The multivariable-adjusted model was adjusted for sex, age, BMI, smoking status, alcohol drinking status, activity level, mean total energy intake, mean Ca intake, as well as Ca supplementation

Multivariable-adjusted relations of OP and radiographic knee OA reveals the multivariable-adjusted association of OP and radiographic knee OA. The outcome suggests a negative association between OP and OA (OR = 0.60, 95 % CI 0.44–0.82, P = 0.001) (Additional file 1).

## Discussion

The present study showed that the overall prevalence of definitive radiographic knee OA was 29.2 % (30.0 % in men, 28.1 % in women). We found lower odds for the appearance of radiographic knee OA and OST in OP patients than in those with normal knees. The prevalence of JSN was higher in patients with osteopenia and OP than in normal subjects.

As far as we know, this is the first study that has evaluated phalangeal BMD in relation to radiographic knee OA with adjustment of various factors for the Chinese population. In line with most previous research findings, this study observed lower odds for the presence of radiographic knee OA and OST in OP patients than in normal knee subjects independent of certain confounding factors, such as sex, age, BMI, smoking, alcohol drinking, activity level, mean total energy intake, mean Ca intake, and Ca supplementation. The proportion of overweight men was higher than that of overweight women (45.8 % vs. 35.5 %, *P* < 0.001), which may be the main reason why the prevalence of knee OA was similar in men and women in this population.

A report from Global Adult Tobacco Survey showed that the overall cigarette smoking rate of the Chinese population >15 years of age is 28.1 %, including 52.9 % of the male population and 2.4 % of the female population [44]. The WHO estimated that, globally, about 53 % of people aged ≥ 15 years have ever consumed alcohol, and that alcohol consumption is generally more prevalent among men [45]. As there is a significantly higher male ratio in the no-OP group than in the OP group, it makes sense that the prevalence of smoking and alcohol drinking is higher in the former group. The prevalence of OA was supposed to be the highest in the no-OP group as people with low BMD are less likely to suffer from OA. However, we observed that the age in the no-OP group was much younger than osteopenia and OP group, which might explain why the prevalence of OA was the lowest in this group.

BMD measurement at the hip and spine with dual-energy X-ray absorptiometry (DXA) is considered the gold standard for diagnosing OP [14, 46]. However, it is a very expensive method that requires frequent calibration to work properly, so it is not always available in developing countries. Using radiographic absorptiometry (RA) (MetriScan) and DXA (Hologic 4500-A; Hologic Inc., Waltham, Mass., USA), Hansen measured the BMD of the intermediate phalanges of the second to fourth fingers, the lumbar spine (L2-L4), and the total hip in 218 men aged 60–74 years (mean 68.8 years). He concluded that RA has moderate ability to identify osteoporotic individuals. RA, however, may be used as a pre-screening tool for men because it could rule out the diagnosis in half the population at little cost [47]. In comparison with DXA as demonstrated by Daniel, this methodology is less expensive, portable, versatile, and accurate for studying changes in the density of materials [48]. The efficacy of phalangeal BMD RA, when using the same device, has been studied by several authors in different cohorts [49–51].

There are several explanations for the lower odds for the presence of radiographic knee OA and OST in OP patients. BMD reflects the quality of bone. A high BMD indicates increased bone quality, which in turn could reduce the fracture risk in patients with OP. Nevitt’s study proved the existence of an association between OSTs and femoral BMD [52]. Both OSTs and bone density can be explained by bone formation. This theory suggests that high BMD is “bone formers” and shows a trend of OSTs formation, whereas low BMD levels are perhaps “bone absorbers” and may cause bone mass loss [53, 54]. Highly increased periarticular bone mass may increase the mechanical stress on cartilage and hence increase the risk of OA [55].

General factors may also play a role in OA as the disease often affects more than a single joint. Dequeker measured iliac crest bone of patients with hand OA and found that elevated serum insulin-like growth factor I (IGF-1) could stimulate OST formation. Therefore, he concluded that insulin might have an anabolic effect on bone, leading to high BMD [56]. In our study, the prevalence of JSN was greater in osteopenia and OP groups than in the no-OP group. This is in accord with Ding’s results, who also found JSN in both hips and knees in association with greater total-hip bone loss [57]. The mechanisms for the associations among JSN, OSTs, and bone loss are unclear. They were all adjusted for BMI, sex, age, activity level, and vitamin D status, suggesting that they may be independent of these common risk factors that are shared by OA and OP.

Summarizing previous studies, high bone mass in OA patients may be caused by multiple factors, including genetic factors, common risk factors, the role of subchondral bone in cartilage damage, actions of growth factors, such as IGF − I and IGF − II, and transforming growth factor β. In this study, with adjustments for age, height, body weight, BMI and so on, the significance was also observed in women with OA of the knee. Therefore, the above factors, in addition to common risk factors (e.g., high body weight or obesity), may contribute to the high BMD in patients with OA of the knee, although their specific relation with BMD have not yet been identified. Further studies may be needed to clarify the uncertainty.

The findings of this study have demonstrated a significant association between elevated metacarpal BMD and radiographic knee OA in the female subgroup, but not in the male subgroup. The reason for the apparent sex difference is still unclear. It may be due to dissimilar risk factor profiles for knee OA. That is, women may be more affected by metabolic factors and men by joint injuries. This study also shows that women with significantly increased BMD—considered to be a protective factor for OP—have a greater chance of developing OA. Higher BMD means that the weight-bearing joints may suffer from heavier impact and pressure, so the cartilage is more likely to be damaged [58]. The present study also indicated a significant association between elevated metacarpal BMD and radiographic knee OA in the normal-weight subgroup, but not in those who are overweight. The reason is not discussed in this study, and further exploration is needed.

Limitations of the present study should also be acknowledged. First, the cross-sectional design of this study precluded causal relationships. Thus, further prospective studies should be carried out to establish a causal association between OA and OP. Second, as our study based on health examination, we only recorded BMD of people who are osteopenia or OP, leaving those with normal BMD unrecorded. But it did not affect data analysis. Another limitation of this study lies in the relatively small sample size of some subgroup analyses. Future research with a larger sample size is needed to confirm the subgroup analysis findings obtained here.

## Conclusions

This study observed lower odds for the presence of radiographic knee OA and OST in OP patients than in normal subjects. The prevalence of JSN was higher in the osteopenia and OP groups than in normal subjects.

## Additional file

Additional file 1: Table S1. Multivariable-adjusted relations of phalangeal BMD with OA, JSN and OST in osteopenia and osteoporosis population (n =1052). (DOCX 14 kb)

## Abbreviations

ADL: Activities of daily life
BMD: Bone mineral density
BMI: Body mass index
Ca: Calcium
CV: Coefficient of variation
DXA: Dual energy X-ray absorptionmetry
FFQ: Food frequency questionnaire
ICC: Intraclass correlation coefficient
IGF: Insulin-like growth factor
K-L: Kellgren-Lawrence
OA: Osteoarthritis
OP: Osteoporosis
QOL: Quality of life
RA: Radiographic absorptiometry
SD: Standard deviations
TGF: Transforming growth factor
SFFQ: Semi-quantitative FFQ
XYHMCS: Xiangya Hospital Health Management Center Study
