# Supplementary material for: Relation between phalangeal bone mineral density and radiographic knee osteoarthritis: a cross-sectional study
Source: BMC Musculoskelet Disord. 2016 Feb 11;17:71. doi: 10.1186/s12891-016-0918-x (PMC4750174; doi:10.1186/s12891-016-0918-x)
Supplement: Additional file 1: Table S1. — Multivariable-adjusted relations of phalangeal BMD with OA, JSN and OST in osteopenia and osteoporosis population (n =1052). (DOCX 14 kb) [file 12891_2016_918_MOESM1_ESM.docx]

**Supplementary table** **1** Multivariable-adjusted relations of phalangeal BMD with OA, JSN and OST in osteopenia and osteoporosis population (n =1052).

|  | Quintiles of BMD | | | | |  |
| --- | --- | --- | --- | --- | --- | --- |
|  | 1 (lowest) | 2 | 3 | 4 | 5 | *P* for trend |
| Participants (n) | 212 | 210 | 210 | 211 | 209 | - |
| Median value of BMD (SD) | 1.12 | 1.44 | 1.83 | 2.33 | 3.14 | - |
| Multivariable-adjusted OR for OA | 1.00 (Reference) | 1.01 (0.67, 1.54) | 0.74 (0.48, 1.14) | 0.85 (0.55, 1.31) | 0.59 (0.37, 0.94) | 0.019 |
| Multivariable-adjusted OR for OST | 1.00 (Reference) | 0.48 (0.25, 0.90) | 0.83 (0.47, 1.45) | 0.65 (0.37, 1.16) | 0.36 (0.20, 0.66) | 0.004 |
| Multivariable-adjusted OR for JSN | 1.00 (Reference) | 0.86 (0.57, 1.31) | 1.00 (0.66, 1.51) | 0.75 (0.49, 1.16) | 1.22 (0.78, 1.90) | 0.418 |

BMD. bone mineral density; OA. Osteoarthritis; OST. Osteophyte; JSN, joint space narrowing.

The BMD were classified into five categories based on the quintile distribution: ≤ 1.29, 1.30-1.61, 1.62-2.07, 2.08 -2.71 and ≥ 2.72 SD. Tests for linear trends were conducted based on logistic regression using a median variable of BMD in each category.

*The multivariable-adjusted model was adjusted for sex, age, BMI, smoking status, alcohol drinking status, activity level, mean total energy intake, mean Ca intake, as well as Ca supplementation.
